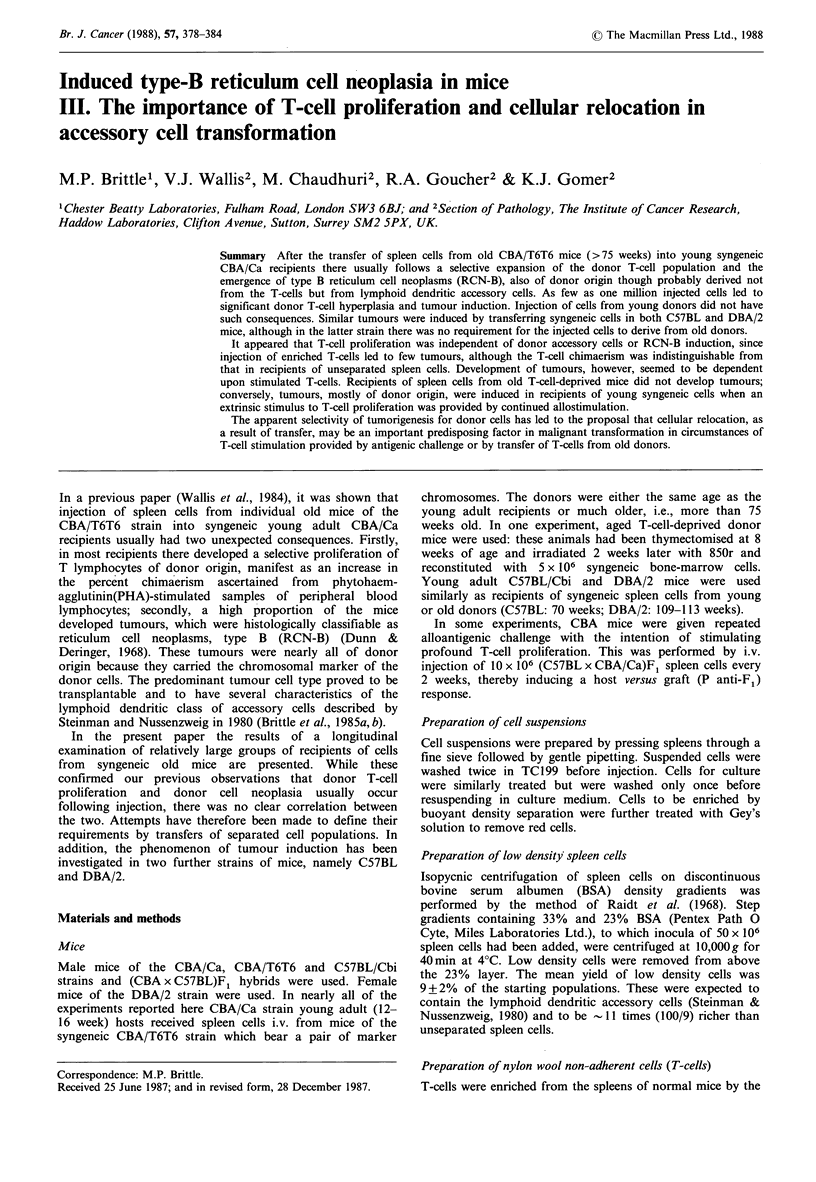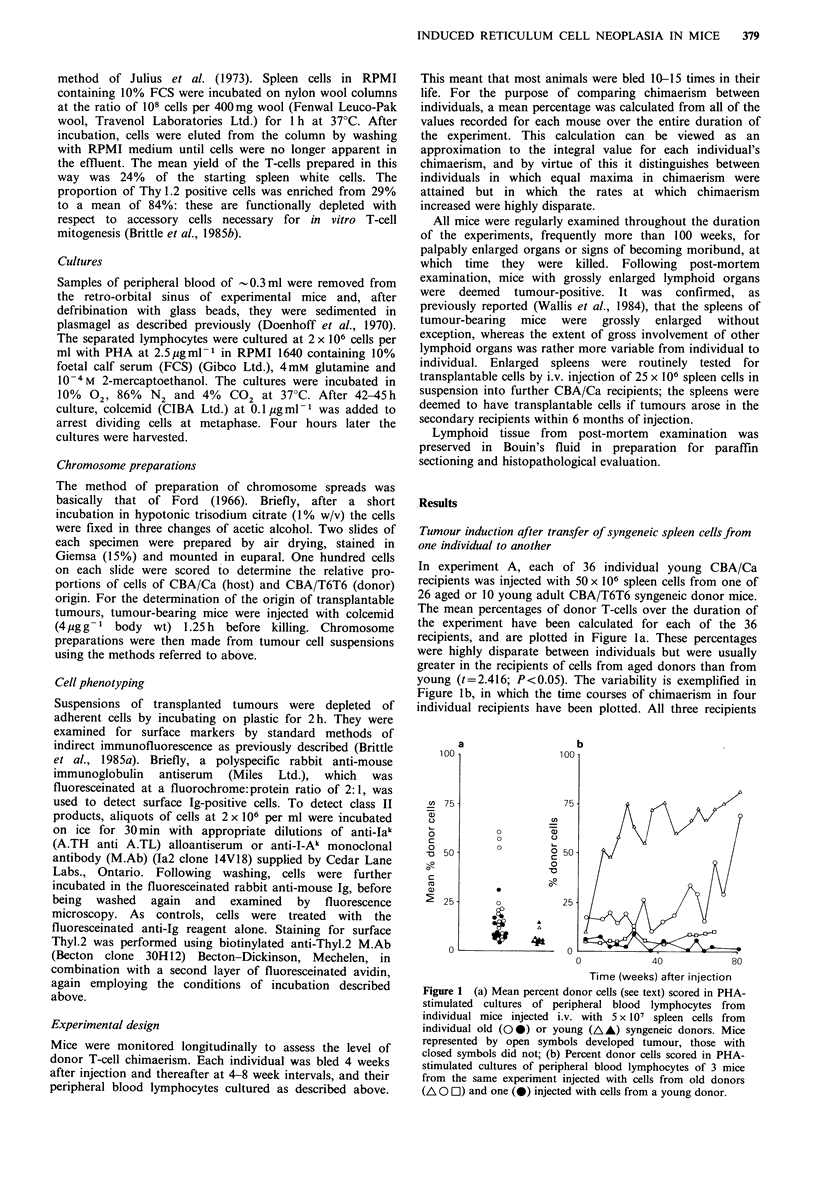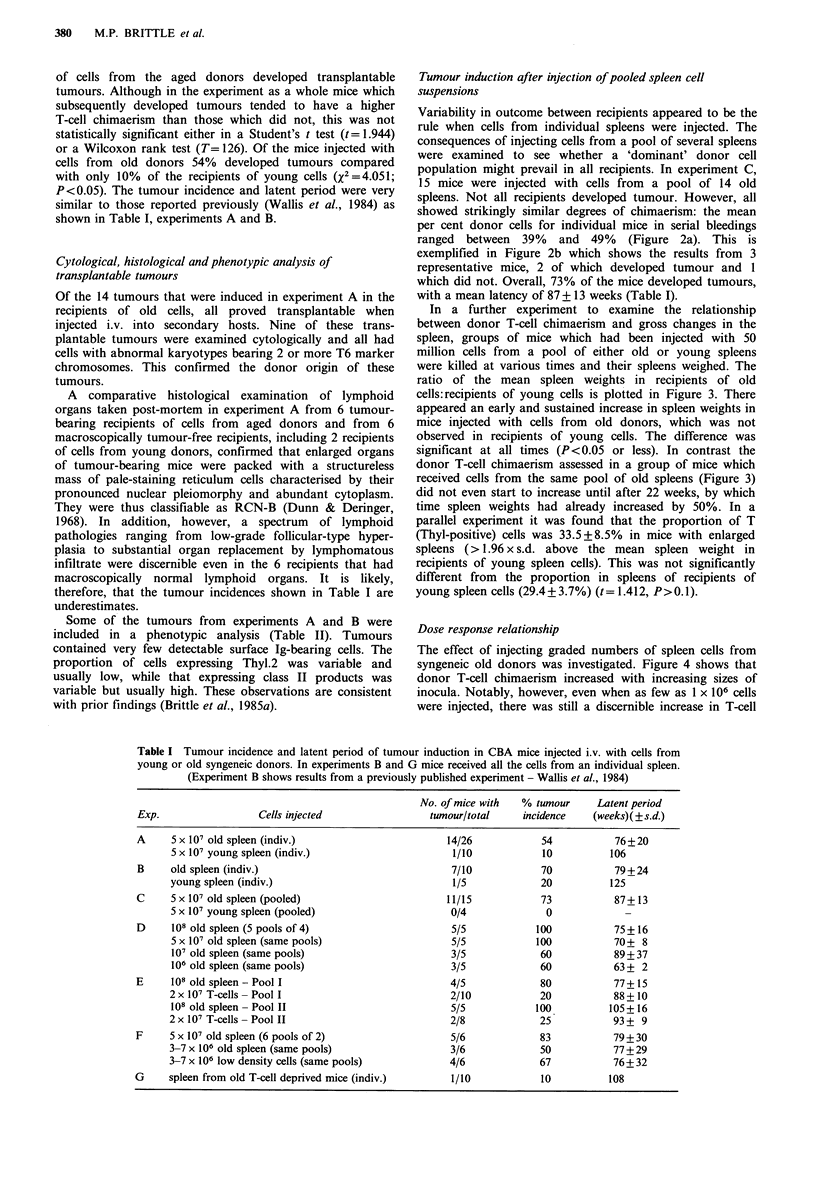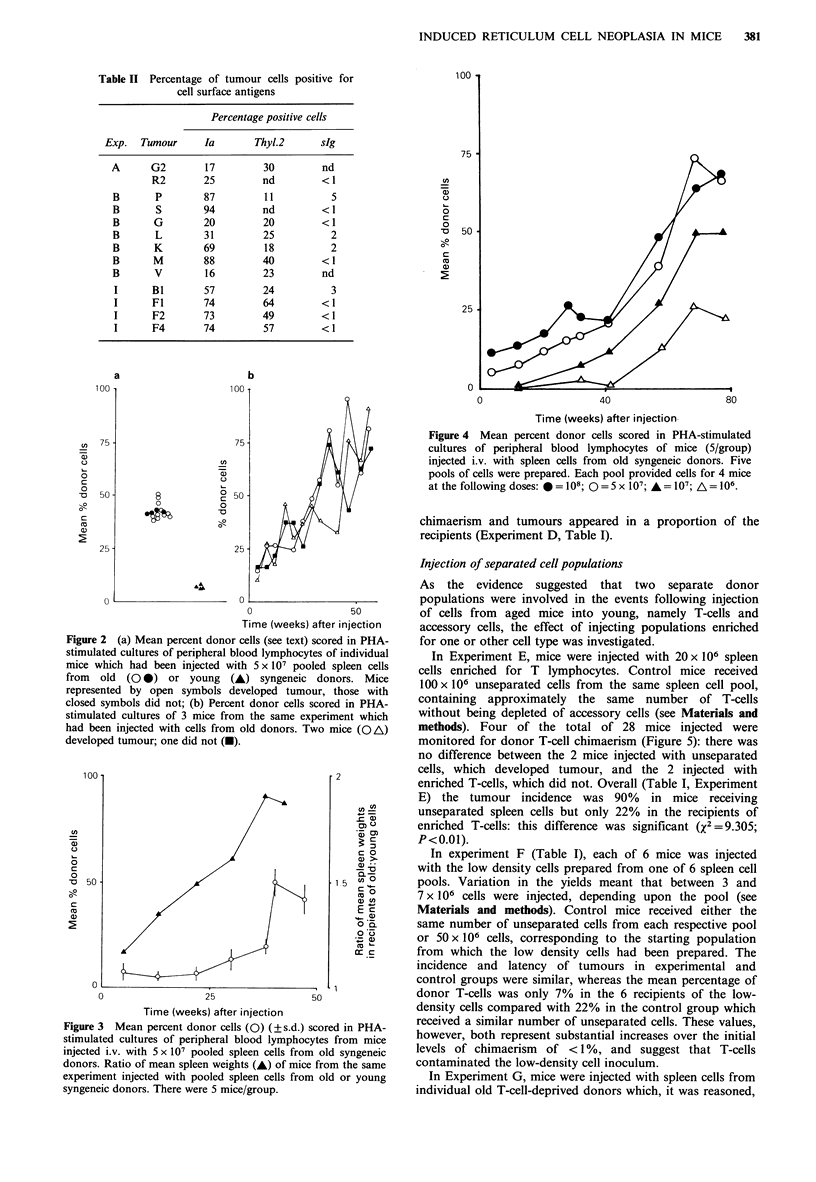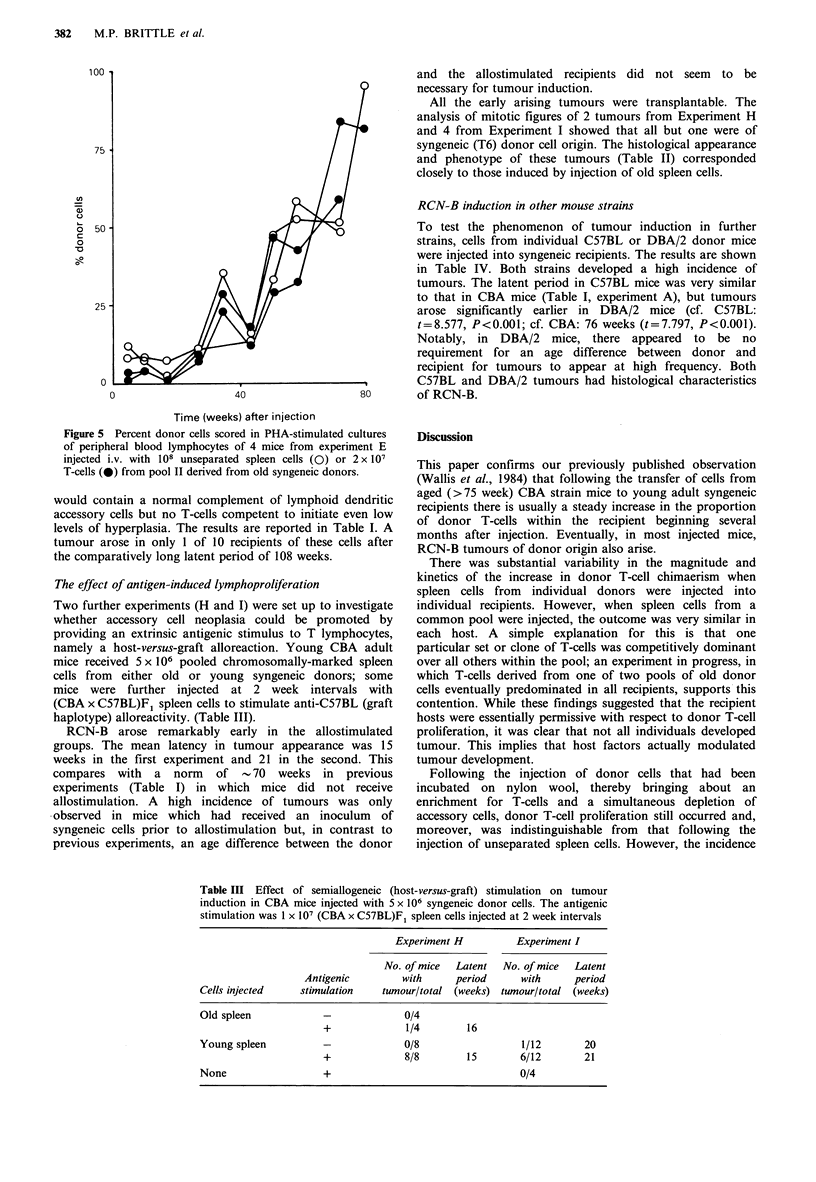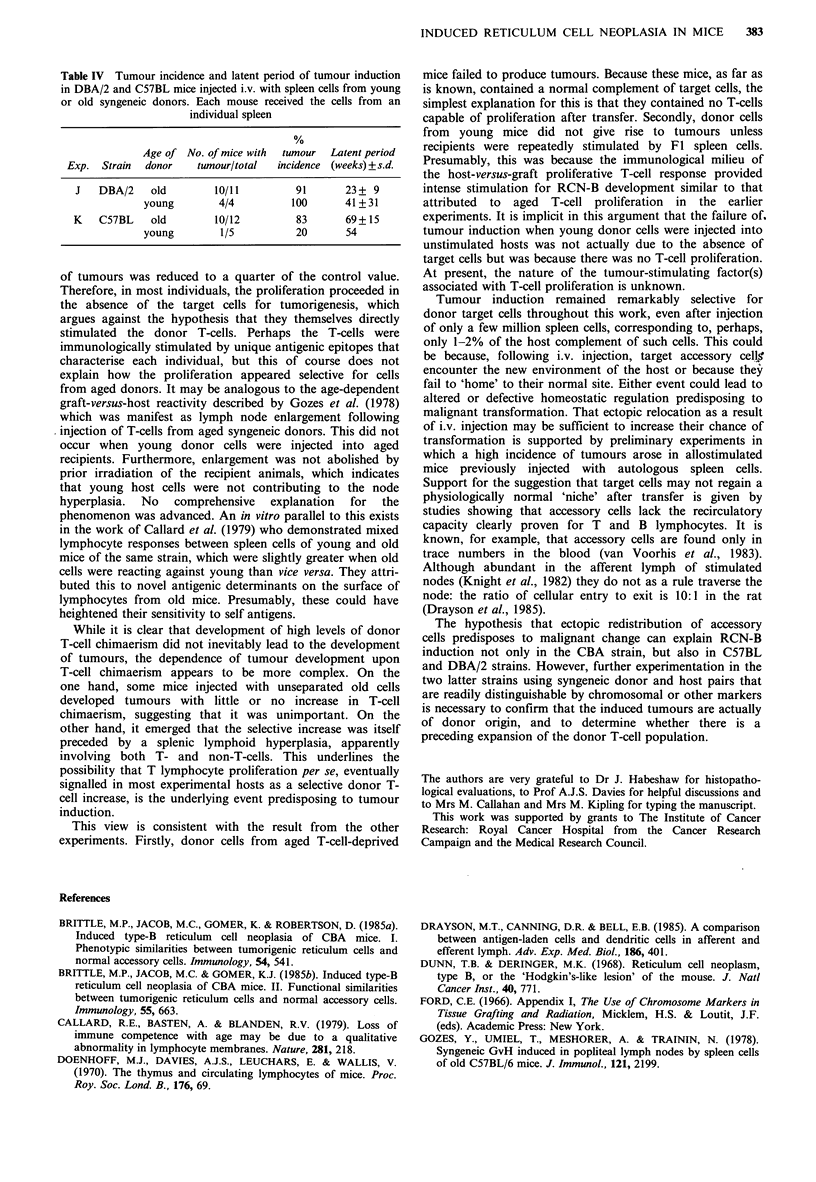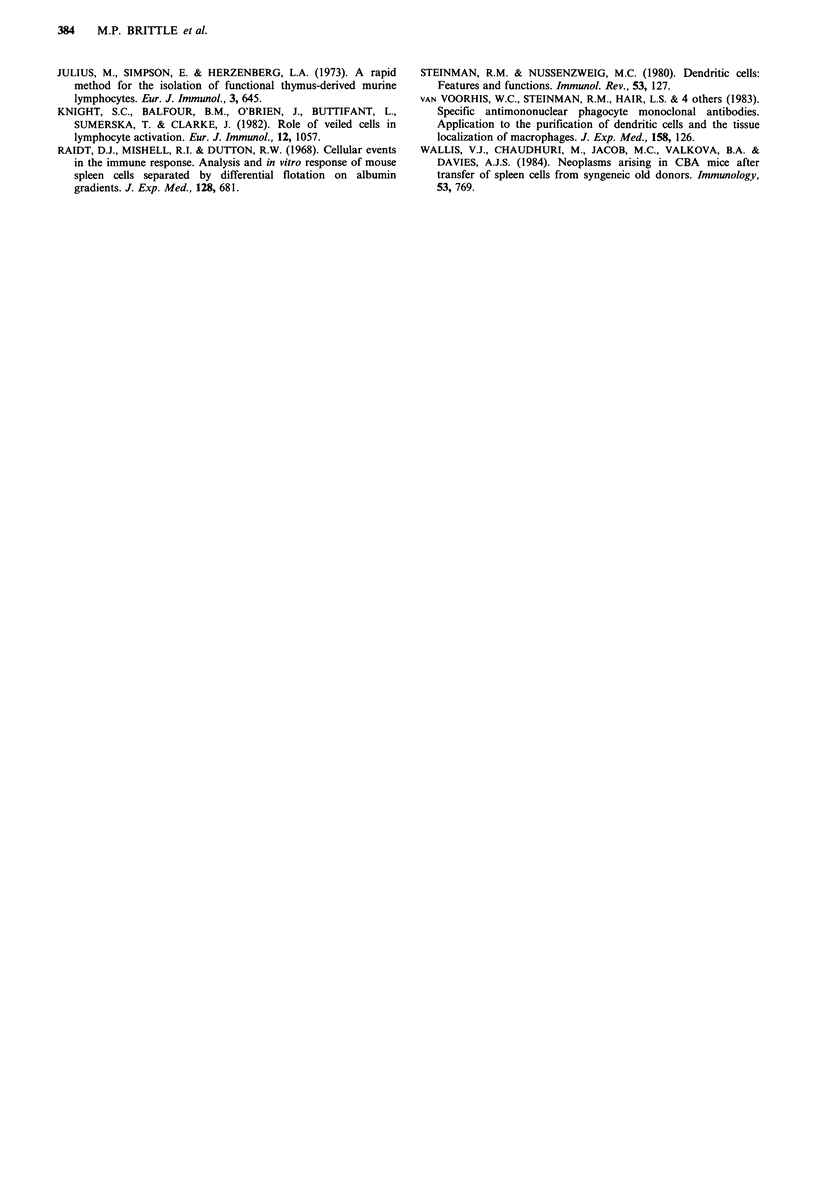# Induced type-B reticulum cell neoplasia in mice III. The importance of T-cell proliferation and cellular relocation in accessory cell transformation.

**DOI:** 10.1038/bjc.1988.86

**Published:** 1988-04

**Authors:** M. P. Brittle, V. J. Wallis, M. Chaudhuri, R. A. Goucher, K. J. Gomer

**Affiliations:** Chester Beatty Laboratories, London, UK.

## Abstract

After the transfer of spleen cells from old CBA/T6T6 mice (greater than 75 weeks) into young syngeneic CBA/Ca recipients there usually follows a selective expansion of the donor T-cell population and the emergence of type B reticulum cell neoplasms (RCN-B), also of donor origin though probably derived not from the T-cells but from lymphoid dendritic accessory cells. As few as one million injected cells led to significant donor T-cell hyperplasia and tumour induction. Injection of cells from young donors did not have such consequences. Similar tumours were induced by transferring syngeneic cells in both C57BL and DBA/2 mice, although in the latter strain there was no requirement for the injected cells to derive from old donors. It appeared that T-cell proliferation was independent of donor accessory cells or RCN-B induction, since injection of enriched T-cells led to few tumours, although the T-cell chimaerism was indistinguishable from that in recipients of unseparated spleen cells. Development of tumours, however, seemed to be dependent upon stimulated T-cells. Recipients of spleen cells from old T-cell-deprived mice did not develop tumours; conversely, tumours, mostly of donor origin, were induced in recipients of young syngeneic cells when an extrinsic stimulus to T-cell proliferation was provided by continued allostimulation. The apparent selectivity of tumorigenesis for donor cells has led to the proposal that cellular relocation, as a result of transfer, may be an important predisposing factor in malignant transformation in circumstances of T-cell stimulation provided by antigenic challenge or by transfer of T-cells from old donors.


Br. J. Cancer (1988), 57, 378–384 © The Macmillan Press Ltd., 1988

### Induced type-B reticulum cell neoplasia in mice
### III. The importance of T-cell proliferation and cellular relocation in accessory cell transformation

M.P. Brittle[1], V.J. Wallis[2], M. Chaudhuri[2], R.A. Goucher[2] & K.J. Gomer[2]

[1]*Chester Beatty Laboratories, Fulham Road, London SW3 6BJ; and* [2]*Section of Pathology, The Institute of Cancer Research, Haddow Laboratories, Clifton Avenue, Sutton, Surrey SM2 5PX, UK.*

**Summary** After the transfer of spleen cells from old CBA/T6T6 mice (>75 weeks) into young syngeneic CBA/Ca recipients there usually follows a selective expansion of the donor T-cell population and the emergence of type B reticulum cell neoplasms (RCN-B), also of donor origin though probably derived not from the T-cells but from lymphoid dendritic accessory cells. As few as one million injected cells led to significant donor T-cell hyperplasia and tumour induction. Injection of cells from young donors did not have such consequences. Similar tumours were induced by transferring syngeneic cells in both C57BL and DBA/2 mice, although in the latter strain there was no requirement for the injected cells to derive from old donors.

It appeared that T-cell proliferation was independent of donor accessory cells or RCN-B induction, since injection of enriched T-cells led to few tumours, although the T-cell chimaerism was indistinguishable from that in recipients of unseparated spleen cells. Development of tumours, however, seemed to be dependent upon stimulated T-cells. Recipients of spleen cells from old T-cell-deprived mice did not develop tumours; conversely, tumours, mostly of donor origin, were induced in recipients of young syngeneic cells when an extrinsic stimulus to T-cell proliferation was provided by continued allostimulation.

The apparent selectivity of tumorigenesis for donor cells has led to the proposal that cellular relocation, as a result of transfer, may be an important predisposing factor in malignant transformation in circumstances of T-cell stimulation provided by antigenic challenge or by transfer of T-cells from old donors.

In a previous paper (Wallis *et al.*, 1984), it was shown that injection of spleen cells from individual old mice of the CBA/T6T6 strain into syngeneic young adult CBA/Ca recipients usually had two unexpected consequences. Firstly, in most recipients there developed a selective proliferation of T lymphocytes of donor origin, manifest as an increase in the percent chimaerism ascertained from phytohaemagglutinin(PHA)-stimulated samples of peripheral blood lymphocytes; secondly, a high proportion of the mice developed tumours, which were histologically classifiable as reticulum cell neoplasms, type B (RCN-B) (Dunn & Deringer, 1968). These tumours were nearly all of donor origin because they carried the chromosomal marker of the donor cells. The predominant tumour cell type proved to be transplantable and to have several characteristics of the lymphoid dendritic class of accessory cells described by Steinman and Nussenzweig in 1980 (Brittle *et al.*, 1985*a*, *b*).

In the present paper the results of a longitudinal examination of relatively large groups of recipients of cells from syngeneic old mice are presented. While these confirmed our previous observations that donor T-cell proliferation and donor cell neoplasia usually occur following injection, there was no clear correlation between the two. Attempts have therefore been made to define their requirements by transfers of separated cell populations. In addition, the phenomenon of tumour induction has been investigated in two further strains of mice, namely C57BL and DBA/2.

### Materials and methods

### *Mice*

Male mice of the CBA/Ca, CBA/T6T6 and C57BL/Cbi strains and (CBA × C57BL)$F_1$ hybrids were used. Female mice of the DBA/2 strain were used. In nearly all of the experiments reported here CBA/Ca strain young adult (12–16 week) hosts received spleen cells i.v. from mice of the syngeneic CBA/T6T6 strain which bear a pair of marker chromosomes. The donors were either the same age as the young adult recipients or much older, i.e., more than 75 weeks old. In one experiment, aged T-cell-deprived donor mice were used: these animals had been thymectomised at 8 weeks of age and irradiated 2 weeks later with 850r and reconstituted with $5 \times 10^6$ syngeneic bone-marrow cells. Young adult C57BL/Cbi and DBA/2 mice were used similarly as recipients of syngeneic spleen cells from young or old donors (C57BL: 70 weeks; DBA/2: 109–113 weeks).

In some experiments, CBA mice were given repeated alloantigenic challenge with the intention of stimulating profound T-cell proliferation. This was performed by i.v. injection of $10 \times 10^6$ (C57BL × CBA/Ca)$F_1$ spleen cells every 2 weeks, thereby inducing a host *versus* graft (P anti-$F_1$) response.

### *Preparation of cell suspensions*

Cell suspensions were prepared by pressing spleens through a fine sieve followed by gentle pipetting. Suspended cells were washed twice in TC199 before injection. Cells for culture were similarly treated but were washed only once before resuspending in culture medium. Cells to be enriched by buoyant density separation were further treated with Gey's solution to remove red cells.

### *Preparation of low density spleen cells*

Isopycnic centrifugation of spleen cells on discontinuous bovine serum albumen (BSA) density gradients was performed by the method of Raidt *et al.* (1968). Step gradients containing 33% and 23% BSA (Pentex Path O Cyte, Miles Laboratories Ltd.), to which inocula of $50 \times 10^6$ spleen cells had been added, were centrifuged at 10,000 *g* for 40 min at 4°C. Low density cells were removed from above the 23% layer. The mean yield of low density cells was $9 \pm 2\%$ of the starting populations. These were expected to contain the lymphoid dendritic accessory cells (Steinman & Nussenzweig, 1980) and to be ∼11 times (100/9) richer than unseparated spleen cells.

### *Preparation of nylon wool non-adherent cells (T-cells)*

T-cells were enriched from the spleens of normal mice by the

Correspondence: M.P. Brittle.
Received 25 June 1987; and in revised form, 28 December 1987.

method of Julius *et al.* (1973). Spleen cells in RPMI containing 10% FCS were incubated on nylon wool columns at the ratio of $10^8$ cells per 400 mg wool (Fenwal Leuco-Pak wool, Travenol Laboratories Ltd.) for 1 h at 37°C. After incubation, cells were eluted from the column by washing with RPMI medium until cells were no longer apparent in the effluent. The mean yield of the T-cells prepared in this way was 24% of the starting spleen white cells. The proportion of Thy 1.2 positive cells was enriched from 29% to a mean of 84%: these are functionally depleted with respect to accessory cells necessary for *in vitro* T-cell mitogenesis (Brittle *et al.*, 1985*b*).

*Cultures*

Samples of peripheral blood of ~0.3 ml were removed from the retro-orbital sinus of experimental mice and, after defribination with glass beads, they were sedimented in plasmagel as described previously (Doenhoff *et al.*, 1970). The separated lymphocytes were cultured at $2 \times 10^6$ cells per ml with PHA at 2.5 $\mu g\,ml^{-1}$ in RPMI 1640 containing 10% foetal calf serum (FCS) (Gibco Ltd.), 4 mM glutamine and $10^{-4}$ M 2-mercaptoethanol. The cultures were incubated in 10% $O_2$, 86% $N_2$ and 4% $CO_2$ at 37°C. After 42–45 h culture, colcemid (CIBA Ltd.) at 0.1 $\mu g\,ml^{-1}$ was added to arrest dividing cells at metaphase. Four hours later the cultures were harvested.

*Chromosome preparations*

The method of preparation of chromosome spreads was basically that of Ford (1966). Briefly, after a short incubation in hypotonic trisodium citrate (1% w/v) the cells were fixed in three changes of acetic alcohol. Two slides of each specimen were prepared by air drying, stained in Giemsa (15%) and mounted in euparal. One hundred cells on each slide were scored to determine the relative proportions of cells of CBA/Ca (host) and CBA/T6T6 (donor) origin. For the determination of the origin of transplantable tumours, tumour-bearing mice were injected with colcemid (4 $\mu g\,g^{-1}$ body wt) 1.25 h before killing. Chromosome preparations were then made from tumour cell suspensions using the methods referred to above.

*Cell phenotyping*

Suspensions of transplanted tumours were depleted of adherent cells by incubating on plastic for 2 h. They were examined for surface markers by standard methods of indirect immunofluorescence as previously described (Brittle *et al.*, 1985*a*). Briefly, a polyspecific rabbit anti-mouse immunoglobulin antiserum (Miles Ltd.), which was fluoresceinated at a fluorochrome:protein ratio of 2:1, was used to detect surface Ig-positive cells. To detect class II products, aliquots of cells at $2 \times 10^6$ per ml were incubated on ice for 30 min with appropriate dilutions of anti-Ia$^k$ (A.TH anti A.TL) alloantiserum or anti-I-A$^k$ monoclonal antibody (M.Ab) (Ia2 clone 14V18) supplied by Cedar Lane Labs., Ontario. Following washing, cells were further incubated in the fluoresceinated rabbit anti-mouse Ig, before being washed again and examined by fluorescence microscopy. As controls, cells were treated with the fluoresceinated anti-Ig reagent alone. Staining for surface Thyl.2 was performed using biotinylated anti-Thyl.2 M.Ab (Becton clone 30H12) Becton–Dickinson, Mechelen, in combination with a second layer of fluoresceinated avidin, again employing the conditions of incubation described above.

*Experimental design*

Mice were monitored longitudinally to assess the level of donor T-cell chimaerism. Each individual was bled 4 weeks after injection and thereafter at 4–8 week intervals, and their peripheral blood lymphocytes cultured as described above. This meant that most animals were bled 10–15 times in their life. For the purpose of comparing chimaerism between individuals, a mean percentage was calculated from all of the values recorded for each mouse over the entire duration of the experiment. This calculation can be viewed as an approximation to the integral value for each individual's chimaerism, and by virtue of this it distinguishes between individuals in which equal maxima in chimaerism were attained but in which the rates at which chimaerism increased were highly disparate.

All mice were regularly examined throughout the duration of the experiments, frequently more than 100 weeks, for palpably enlarged organs or signs of becoming moribund, at which time they were killed. Following post-mortem examination, mice with grossly enlarged lymphoid organs were deemed tumour-positive. It was confirmed, as previously reported (Wallis *et al.*, 1984), that the spleens of tumour-bearing mice were grossly enlarged without exception, whereas the extent of gross involvement of other lymphoid organs was rather more variable from individual to individual. Enlarged spleens were routinely tested for transplantable cells by i.v. injection of $25 \times 10^6$ spleen cells in suspension into further CBA/Ca recipients; the spleens were deemed to have transplantable cells if tumours arose in the secondary recipients within 6 months of injection.

Lymphoid tissue from post-mortem examination was preserved in Bouin's fluid in preparation for paraffin sectioning and histopathological evaluation.

### Results

*Tumour induction after transfer of syngeneic spleen cells from one individual to another*

In experiment A, each of 36 individual young CBA/Ca recipients was injected with $50 \times 10^6$ spleen cells from one of 26 aged or 10 young adult CBA/T6T6 syngeneic donor mice. The mean percentages of donor T-cells over the duration of the experiment have been calculated for each of the 36 recipients, and are plotted in Figure 1a. These percentages were highly disparate between individuals but were usually greater in the recipients of cells from aged donors than from young ($t = 2.416$; $P < 0.05$). The variability is exemplified in Figure 1b, in which the time courses of chimaerism in four individual recipients have been plotted. All three recipients

**Figure 1** (a) Mean percent donor cells (see text) scored in PHA-stimulated cultures of peripheral blood lymphocytes from individual mice injected i.v. with $5 \times 10^7$ spleen cells from individual old (○●) or young (△▲) syngeneic donors. Mice represented by open symbols developed tumour, those with closed symbols did not; (b) Percent donor cells scored in PHA-stimulated cultures of peripheral blood lymphocytes of 3 mice from the same experiment injected with cells from old donors (△○□) and one (●) injected with cells from a young donor.

of cells from the aged donors developed transplantable tumours. Although in the experiment as a whole mice which subsequently developed tumours tended to have a higher T-cell chimaerism than those which did not, this was not statistically significant either in a Student's $t$ test ($t=1.944$) or a Wilcoxon rank test ($T=126$). Of the mice injected with cells from old donors 54% developed tumours compared with only 10% of the recipients of young cells ($\chi^2=4.051$; $P<0.05$). The tumour incidence and latent period were very similar to those reported previously (Wallis *et al.*, 1984) as shown in Table I, experiments A and B.

### *Cytological, histological and phenotypic analysis of transplantable tumours*

Of the 14 tumours that were induced in experiment A in the recipients of old cells, all proved transplantable when injected i.v. into secondary hosts. Nine of these transplantable tumours were examined cytologically and all had cells with abnormal karyotypes bearing 2 or more T6 marker chromosomes. This confirmed the donor origin of these tumours.

A comparative histological examination of lymphoid organs taken post-mortem in experiment A from 6 tumour-bearing recipients of cells from aged donors and from 6 macroscopically tumour-free recipients, including 2 recipients of cells from young donors, confirmed that enlarged organs of tumour-bearing mice were packed with a structureless mass of pale-staining reticulum cells characterised by their pronounced nuclear pleiomorphy and abundant cytoplasm. They were thus classifiable as RCN-B (Dunn & Deringer, 1968). In addition, however, a spectrum of lymphoid pathologies ranging from low-grade follicular-type hyperplasia to substantial organ replacement by lymphomatous infiltrate were discernible even in the 6 recipients that had macroscopically normal lymphoid organs. It is likely, therefore, that the tumour incidences shown in Table I are underestimates.

Some of the tumours from experiments A and B were included in a phenotypic analysis (Table II). Tumours contained very few detectable surface Ig-bearing cells. The proportion of cells expressing Thyl.2 was variable and usually low, while that expressing class II products was variable but usually high. These observations are consistent with prior findings (Brittle *et al.*, 1985*a*).

### *Tumour induction after injection of pooled spleen cell suspensions*

Variability in outcome between recipients appeared to be the rule when cells from individual spleens were injected. The consequences of injecting cells from a pool of several spleens were examined to see whether a 'dominant' donor cell population might prevail in all recipients. In experiment C, 15 mice were injected with cells from a pool of 14 old spleens. Not all recipients developed tumour. However, all showed strikingly similar degrees of chimaerism: the mean per cent donor cells for individual mice in serial bleedings ranged between 39% and 49% (Figure 2a). This is exemplified in Figure 2b which shows the results from 3 representative mice, 2 of which developed tumour and 1 which did not. Overall, 73% of the mice developed tumours, with a mean latency of $87\pm13$ weeks (Table I).

In a further experiment to examine the relationship between donor T-cell chimaerism and gross changes in the spleen, groups of mice which had been injected with 50 million cells from a pool of either old or young spleens were killed at various times and their spleens weighed. The ratio of the mean spleen weights in recipients of old cells:recipients of young cells is plotted in Figure 3. There appeared an early and sustained increase in spleen weights in mice injected with cells from old donors, which was not observed in recipients of young cells. The difference was significant at all times ($P<0.05$ or less). In contrast the donor T-cell chimaerism assessed in a group of mice which received cells from the same pool of old spleens (Figure 3) did not even start to increase until after 22 weeks, by which time spleen weights had already increased by 50%. In a parallel experiment it was found that the proportion of T (Thyl-positive) cells was $33.5\pm8.5\%$ in mice with enlarged spleens ($>1.96\times$ s.d. above the mean spleen weight in recipients of young spleen cells). This was not significantly different from the proportion in spleens of recipients of young spleen cells ($29.4\pm3.7\%$) ($t=1.412$, $P>0.1$).

### *Dose response relationship*

The effect of injecting graded numbers of spleen cells from syngeneic old donors was investigated. Figure 4 shows that donor T-cell chimaerism increased with increasing sizes of inocula. Notably, however, even when as few as $1\times10^6$ cells were injected, there was still a discernible increase in T-cell

**Table I** Tumour incidence and latent period of tumour induction in CBA mice injected i.v. with cells from young or old syngeneic donors. In experiments B and G mice received all the cells from an individual spleen. (Experiment B shows results from a previously published experiment – Wallis *et al.*, 1984)

| *Exp.* | *Cells injected* | *No. of mice with tumour/total* | *% tumour incidence* | *Latent period (weeks)(±s.d.)* |
|---|---|---|---|---|
| A | $5\times10^7$ old spleen (indiv.) | 14/26 | 54 | $76\pm20$ |
| | $5\times10^7$ young spleen (indiv.) | 1/10 | 10 | 106 |
| B | old spleen (indiv.) | 7/10 | 70 | $79\pm24$ |
| | young spleen (indiv.) | 1/5 | 20 | 125 |
| C | $5\times10^7$ old spleen (pooled) | 11/15 | 73 | $87\pm13$ |
| | $5\times10^7$ young spleen (pooled) | 0/4 | 0 | – |
| D | $10^8$ old spleen (5 pools of 4) | 5/5 | 100 | $75\pm16$ |
| | $5\times10^7$ old spleen (same pools) | 5/5 | 100 | $70\pm8$ |
| | $10^7$ old spleen (same pools) | 3/5 | 60 | $89\pm37$ |
| | $10^6$ old spleen (same pools) | 3/5 | 60 | $63\pm2$ |
| E | $10^8$ old spleen – Pool I | 4/5 | 80 | $77\pm15$ |
| | $2\times10^7$ T-cells – Pool I | 2/10 | 20 | $88\pm10$ |
| | $10^8$ old spleen – Pool II | 5/5 | 100 | $105\pm16$ |
| | $2\times10^7$ T-cells – Pool II | 2/8 | 25 | $93\pm9$ |
| F | $5\times10^7$ old spleen (6 pools of 2) | 5/6 | 83 | $79\pm30$ |
| | $3–7\times10^6$ old spleen (same pools) | 3/6 | 50 | $77\pm29$ |
| | $3–7\times10^6$ low density cells (same pools) | 4/6 | 67 | $76\pm32$ |
| G | spleen from old T-cell deprived mice (indiv.) | 1/10 | 10 | 108 |

**Table II** Percentage of tumour cells positive for cell surface antigens

| | | *Percentage positive cells* | | |
|---|---|---|---|---|
| *Exp.* | *Tumour* | *Ia* | *Thyl.2* | *sIg* |
| A | G2 | 17 | 30 | nd |
| | R2 | 25 | nd | <1 |
| B | P | 87 | 11 | 5 |
| B | S | 94 | nd | <1 |
| B | G | 20 | 20 | <1 |
| B | L | 31 | 25 | 2 |
| B | K | 69 | 18 | 2 |
| B | M | 88 | 40 | <1 |
| B | V | 16 | 23 | nd |
| I | B1 | 57 | 24 | 3 |
| I | F1 | 74 | 64 | <1 |
| I | F2 | 73 | 49 | <1 |
| I | F4 | 74 | 57 | <1 |

**Figure 2** (a) Mean percent donor cells (see text) scored in PHA-stimulated cultures of peripheral blood lymphocytes of individual mice which had been injected with $5 \times 10^7$ pooled spleen cells from old (○●) or young (▲) syngeneic donors. Mice represented by open symbols developed tumour, those with closed symbols did not; (b) Percent donor cells scored in PHA-stimulated cultures of 3 mice from the same experiment which had been injected with cells from old donors. Two mice (○△) developed tumour; one did not (■).

**Figure 3** Mean percent donor cells (○) (±s.d.) scored in PHA-stimulated cultures of peripheral blood lymphocytes from mice injected i.v. with $5 \times 10^7$ pooled spleen cells from old syngeneic donors. Ratio of mean spleen weights (▲) of mice from the same experiment injected with pooled spleen cells from old or young syngeneic donors. There were 5 mice/group.

**Figure 4** Mean percent donor cells scored in PHA-stimulated cultures of peripheral blood lymphocytes of mice (5/group) injected i.v. with spleen cells from old syngeneic donors. Five pools of cells were prepared. Each pool provided cells for 4 mice at the following doses: ● $= 10^8$; ○ $= 5 \times 10^7$; ▲ $= 10^7$; △ $= 10^6$.

chimaerism and tumours appeared in a proportion of the recipients (Experiment D, Table I).

### *Injection of separated cell populations*

As the evidence suggested that two separate donor populations were involved in the events following injection of cells from aged mice into young, namely T-cells and accessory cells, the effect of injecting populations enriched for one or other cell type was investigated.

In Experiment E, mice were injected with $20 \times 10^6$ spleen cells enriched for T lymphocytes. Control mice received $100 \times 10^6$ unseparated cells from the same spleen cell pool, containing approximately the same number of T-cells without being depleted of accessory cells (see **Materials and methods**). Four of the total of 28 mice injected were monitored for donor T-cell chimaerism (Figure 5): there was no difference between the 2 mice injected with unseparated cells, which developed tumour, and the 2 injected with enriched T-cells, which did not. Overall (Table I, Experiment E) the tumour incidence was 90% in mice receiving unseparated spleen cells but only 22% in the recipients of enriched T-cells: this difference was significant ($\chi^2 = 9.305$; $P < 0.01$).

In experiment F (Table I), each of 6 mice was injected with the low density cells prepared from one of 6 spleen cell pools. Variation in the yields meant that between 3 and $7 \times 10^6$ cells were injected, depending upon the pool (see **Materials and methods**). Control mice received either the same number of unseparated cells from each respective pool or $50 \times 10^6$ cells, corresponding to the starting population from which the low density cells had been prepared. The incidence and latency of tumours in experimental and control groups were similar, whereas the mean percentage of donor T-cells was only 7% in the 6 recipients of the low-density cells compared with 22% in the control group which received a similar number of unseparated cells. These values, however, both represent substantial increases over the initial levels of chimaerism of $< 1\%$, and suggest that T-cells contaminated the low-density cell inoculum.

In Experiment G, mice were injected with spleen cells from individual old T-cell-deprived donors which, it was reasoned,

**Figure 5** Percent donor cells scored in PHA-stimulated cultures of peripheral blood lymphocytes of 4 mice from experiment E injected i.v. with $10^8$ unseparated spleen cells (○) or $2 \times 10^7$ T-cells (●) from pool II derived from old syngeneic donors.

would contain a normal complement of lymphoid dendritic accessory cells but no T-cells competent to initiate even low levels of hyperplasia. The results are reported in Table I. A tumour arose in only 1 of 10 recipients of these cells after the comparatively long latent period of 108 weeks.

*The effect of antigen-induced lymphoproliferation*

Two further experiments (H and I) were set up to investigate whether accessory cell neoplasia could be promoted by providing an extrinsic antigenic stimulus to T lymphocytes, namely a host-*versus*-graft alloreaction. Young CBA adult mice received $5 \times 10^6$ pooled chromosomally-marked spleen cells from either old or young syngeneic donors; some mice were further injected at 2 week intervals with (CBA x C57BL)$F_1$ spleen cells to stimulate anti-C57BL (graft haplotype) alloreactivity. (Table III).

RCN-B arose remarkably early in the allostimulated groups. The mean latency in tumour appearance was 15 weeks in the first experiment and 21 in the second. This compares with a norm of ~70 weeks in previous experiments (Table I) in which mice did not receive allostimulation. A high incidence of tumours was only observed in mice which had received an inoculum of syngeneic cells prior to allostimulation but, in contrast to previous experiments, an age difference between the donor and the allostimulated recipients did not seem to be necessary for tumour induction.

All the early arising tumours were transplantable. The analysis of mitotic figures of 2 tumours from Experiment H and 4 from Experiment I showed that all but one were of syngeneic (T6) donor cell origin. The histological appearance and phenotype of these tumours (Table II) corresponded closely to those induced by injection of old spleen cells.

*RCN-B induction in other mouse strains*

To test the phenomenon of tumour induction in further strains, cells from individual C57BL or DBA/2 donor mice were injected into syngeneic recipients. The results are shown in Table IV. Both strains developed a high incidence of tumours. The latent period in C57BL mice was very similar to that in CBA mice (Table I, experiment A), but tumours arose significantly earlier in DBA/2 mice (cf. C57BL: $t=8.577$, $P<0.001$; cf. CBA: 76 weeks ($t=7.797$, $P<0.001$). Notably, in DBA/2 mice, there appeared to be no requirement for an age difference between donor and recipient for tumours to appear at high frequency. Both C57BL and DBA/2 tumours had histological characteristics of RCN-B.

### Discussion

This paper confirms our previously published observation (Wallis *et al.*, 1984) that following the transfer of cells from aged (>75 week) CBA strain mice to young adult syngeneic recipients there is usually a steady increase in the proportion of donor T-cells within the recipient beginning several months after injection. Eventually, in most injected mice, RCN-B tumours of donor origin also arise.

There was substantial variability in the magnitude and kinetics of the increase in donor T-cell chimaerism when spleen cells from individual donors were injected into individual recipients. However, when spleen cells from a common pool were injected, the outcome was very similar in each host. A simple explanation for this is that one particular set or clone of T-cells was competitively dominant over all others within the pool; an experiment in progress, in which T-cells derived from one of two pools of old donor cells eventually predominated in all recipients, supports this contention. While these findings suggested that the recipient hosts were essentially permissive with respect to donor T-cell proliferation, it was clear that not all individuals developed tumour. This implies that host factors actually modulated tumour development.

Following the injection of donor cells that had been incubated on nylon wool, thereby bringing about an enrichment for T-cells and a simultaneous depletion of accessory cells, donor T-cell proliferation still occurred and, moreover, was indistinguishable from that following the injection of unseparated spleen cells. However, the incidence

**Table III** Effect of semiallogeneic (host-*versus*-graft) stimulation on tumour induction in CBA mice injected with $5 \times 10^6$ syngeneic donor cells. The antigenic stimulation was $1 \times 10^7$ (CBA x C57BL)$F_1$ spleen cells injected at 2 week intervals

| | | *Experiment H* | | *Experiment I* | |
|---|---|---|---|---|---|
| *Cells injected* | *Antigenic stimulation* | *No. of mice with tumour/total* | *Latent period (weeks)* | *No. of mice with tumour/total* | *Latent period (weeks)* |
| Old spleen | – | 0/4 | | | |
| | + | 1/4 | 16 | | |
| Young spleen | – | 0/8 | | 1/12 | 20 |
| | + | 8/8 | 15 | 6/12 | 21 |
| None | + | | | 0/4 | |

**Table IV** Tumour incidence and latent period of tumour induction in DBA/2 and C57BL mice injected i.v. with spleen cells from young or old syngeneic donors. Each mouse received the cells from an individual spleen

| *Exp.* | *Strain* | *Age of donor* | *No. of mice with tumour/total* | % *tumour incidence* | *Latent period (weeks)* ± *s.d.* |
|---|---|---|---|---|---|
| J | DBA/2 | old | 10/11 | 91 | 23 ± 9 |
| | | young | 4/4 | 100 | 41 ± 31 |
| K | C57BL | old | 10/12 | 83 | 69 ± 15 |
| | | young | 1/5 | 20 | 54 |

of tumours was reduced to a quarter of the control value. Therefore, in most individuals, the proliferation proceeded in the absence of the target cells for tumorigenesis, which argues against the hypothesis that they themselves directly stimulated the donor T-cells. Perhaps the T-cells were immunologically stimulated by unique antigenic epitopes that characterise each individual, but this of course does not explain how the proliferation appeared selective for cells from aged donors. It may be analogous to the age-dependent graft-*versus*-host reactivity described by Gozes *et al.* (1978) which was manifest as lymph node enlargement following injection of T-cells from aged syngeneic donors. This did not occur when young donor cells were injected into aged recipients. Furthermore, enlargement was not abolished by prior irradiation of the recipient animals, which indicates that young host cells were not contributing to the node hyperplasia. No comprehensive explanation for the phenomenon was advanced. An *in vitro* parallel to this exists in the work of Callard *et al.* (1979) who demonstrated mixed lymphocyte responses between spleen cells of young and old mice of the same strain, which were slightly greater when old cells were reacting against young than *vice versa*. They attributed this to novel antigenic determinants on the surface of lymphocytes from old mice. Presumably, these could have heightened their sensitivity to self antigens.

While it is clear that development of high levels of donor T-cell chimaerism did not inevitably lead to the development of tumours, the dependence of tumour development upon T-cell chimaerism appears to be more complex. On the one hand, some mice injected with unseparated old cells developed tumours with little or no increase in T-cell chimaerism, suggesting that it was unimportant. On the other hand, it emerged that the selective increase was itself preceded by a splenic lymphoid hyperplasia, apparently involving both T- and non-T-cells. This underlines the possibility that T lymphocyte proliferation *per se*, eventually signalled in most experimental hosts as a selective donor T-cell increase, is the underlying event predisposing to tumour induction.

This view is consistent with the result from the other experiments. Firstly, donor cells from aged T-cell-deprived mice failed to produce tumours. Because these mice, as far as is known, contained a normal complement of target cells, the simplest explanation for this is that they contained no T-cells capable of proliferation after transfer. Secondly, donor cells from young mice did not give rise to tumours unless recipients were repeatedly stimulated by F1 spleen cells. Presumably, this was because the immunological milieu of the host-*versus*-graft proliferative T-cell response provided intense stimulation for RCN-B development similar to that attributed to aged T-cell proliferation in the earlier experiments. It is implicit in this argument that the failure of tumour induction when young donor cells were injected into unstimulated hosts was not actually due to the absence of target cells but was because there was no T-cell proliferation. At present, the nature of the tumour-stimulating factor(s) associated with T-cell proliferation is unknown.

Tumour induction remained remarkably selective for donor target cells throughout this work, even after injection of only a few million spleen cells, corresponding to, perhaps, only 1–2% of the host complement of such cells. This could be because, following i.v. injection, target accessory cells encounter the new environment of the host or because they fail to 'home' to their normal site. Either event could lead to altered or defective homeostatic regulation predisposing to malignant transformation. That ectopic relocation as a result of i.v. injection may be sufficient to increase their chance of transformation is supported by preliminary experiments in which a high incidence of tumours arose in allostimulated mice previously injected with autologous spleen cells. Support for the suggestion that target cells may not regain a physiologically normal 'niche' after transfer is given by studies showing that accessory cells lack the recirculatory capacity clearly proven for T and B lymphocytes. It is known, for example, that accessory cells are found only in trace numbers in the blood (van Voorhis *et al.*, 1983). Although abundant in the afferent lymph of stimulated nodes (Knight *et al.*, 1982) they do not as a rule traverse the node: the ratio of cellular entry to exit is 10:1 in the rat (Drayson *et al.*, 1985).

The hypothesis that ectopic redistribution of accessory cells predisposes to malignant change can explain RCN-B induction not only in the CBA strain, but also in C57BL and DBA/2 strains. However, further experimentation in the two latter strains using syngeneic donor and host pairs that are readily distinguishable by chromosomal or other markers is necessary to confirm that the induced tumours are actually of donor origin, and to determine whether there is a preceding expansion of the donor T-cell population.

The authors are very grateful to Dr J. Habeshaw for histopathological evaluations, to Prof A.J.S. Davies for helpful discussions and to Mrs M. Callahan and Mrs M. Kipling for typing the manuscript.

This work was supported by grants to The Institute of Cancer Research: Royal Cancer Hospital from the Cancer Research Campaign and the Medical Research Council.

### References

BRITTLE, M.P., JACOB, M.C., GOMER, K. & ROBERTSON, D. (1985*a*). Induced type-B reticulum cell neoplasia of CBA mice. I. Phenotypic similarities between tumorigenic reticulum cells and normal accessory cells. *Immunology*, **54**, 541.

BRITTLE, M.P., JACOB, M.C. & GOMER, K.J. (1985*b*). Induced type-B reticulum cell neoplasia of CBA mice. II. Functional similarities between tumorigenic reticulum cells and normal accessory cells. *Immunology*, **55**, 663.

CALLARD, R.E., BASTEN, A. & BLANDEN, R.V. (1979). Loss of immune competence with age may be due to a qualitative abnormality in lymphocyte membranes. *Nature*, **281**, 218.

DOENHOFF, M.J., DAVIES, A.J.S., LEUCHARS, E. & WALLIS, V. (1970). The thymus and circulating lymphocytes of mice. *Proc. Roy. Soc. Lond. B.*, **176**, 69.

DRAYSON, M.T., CANNING, D.R. & BELL, E.B. (1985). A comparison between antigen-laden cells and dendritic cells in afferent and efferent lymph. *Adv. Exp. Med. Biol.*, **186**, 401.

DUNN, T.B. & DERINGER, M.K. (1968). Reticulum cell neoplasm, type B, or the 'Hodgkin's-like lesion' of the mouse. *J. Natl Cancer Inst.*, **40**, 771.

FORD, C.E. (1966). Appendix I, *The Use of Chromosome Markers in Tissue Grafting and Radiation*, Micklem, H.S. & Loutit, J.F. (eds). Academic Press: New York.

GOZES, Y., UMIEL, T., MESHORER, A. & TRAININ, N. (1978). Syngeneic GvH induced in popliteal lymph nodes by spleen cells of old C57BL/6 mice. *J. Immunol.*, **121**, 2199.

JULIUS, M., SIMPSON, E. & HERZENBERG, L.A. (1973). A rapid method for the isolation of functional thymus-derived murine lymphocytes. *Eur. J. Immunol.*, **3**, 645.

KNIGHT, S.C., BALFOUR, B.M., O'BRIEN, J., BUTTIFANT, L., SUMERSKA, T. & CLARKE, J. (1982). Role of veiled cells in lymphocyte activation. *Eur. J. Immunol.*, **12**, 1057.

RAIDT, D.J., MISHELL, R.I. & DUTTON, R.W. (1968). Cellular events in the immune response. Analysis and *in vitro* response of mouse spleen cells separated by differential flotation on albumin gradients. *J. Exp. Med.*, **128**, 681.

STEINMAN, R.M. & NUSSENZWEIG, M.C. (1980). Dendritic cells: Features and functions. *Immunol. Rev.*, **53**, 127.

VAN VOORHIS, W.C., STEINMAN, R.M., HAIR, L.S. & 4 others (1983). Specific antimononuclear phagocyte monoclonal antibodies. Application to the purification of dendritic cells and the tissue localization of macrophages. *J. Exp. Med.*, **158**, 126.

WALLIS, V.J., CHAUDHURI, M., JACOB, M.C., VALKOVA, B.A. & DAVIES, A.J.S. (1984). Neoplasms arising in CBA mice after transfer of spleen cells from syngeneic old donors. *Immunology*, **53**, 769.